# Correction

**DOI:** 10.1080/10872981.2024.2334472

**Published:** 2024-03-29

**Article title**: Medical student non-modifiable risk factors and USMLE Step 1 exam score

**Authors**: Jenna M. Davison, Margot B. Taylor and Tracy N. Bumsted

**Journal**: *Medical Education Online*

**Bibliometrics**: Volume 29, Number 01, pages 1-4

DOI: https://doi.org/10.1080/10872981.2024.2327818

It has been noted by the authors that the third column of table 1 reads “ Met Threshold Step 1 score of 220 (%)”, however it should read “Not Met Threshold Step 1 score of 220 (%)”. The corrected table 1 has been placed below. This correction has not changed the description, interpretation, or the original conclusions of the article. The authors apologize for any inconvenience caused.

**Table 1.** Descriptive statistics non-modifiable risk factors and analysis with unadjusted risk ratios.

Non-modifiable Risk Factor	Sample number n (%)	Not Met Threshold Step 1score of 220 (%)	Unadjusted Risk Ratios(95% CI)	p-value
ACE score <3 ≥3	59 (72.8) 22 (28.2)	9 (15.2) 12 (54.5)	– 3.58 (1.75-7.29)	– <0.01
Representation in Medicine by Race or Ethnicity ORiM URiM	70 (86.4) 11 (13.6)	16 (22.9) 5 (45.5)	– 1.99 (0.91 -4.32)	– 0.11
Sex Female Male	53 (65.4) 28 (34.6)	15 (28.3) 6 (21.4)	– 1.32 (0.58-3.02)	– 0.50
Gender Man Woman Non-binary or Genderqueer	28 (34.6) 48 (59.3) 5 (6.1)	6 (21.4) 14 (29.1) 1 (20.0)	– 1.36 (0.60-3.14) 0.93 (0.14-6.18)	– 0.46 0.94
Discrimination Experience No Yes	51 (63.0) 30 (37.0)	10 (19.6) 11 (36.7)	– 1.87 (0.90-3.87)	– 0.09
Living Location when <18 Urban Rural	59 (72.8) 22 (27.2)	14 (23.7) 7 (31.8)	– 1.34 (0.62-2.88)	– 0.46
Average Household Income when <18 $0-74,999 $75,000-$99,999 $100,000-$149,999 $150,000-$249,999 $250,000 and up	12 (14.8) 12 (14.8) 22 (27.2) 15 (18.5) 8 (9.7)	8 (66.7) 4 (33.3) 4 (18.2) 2 (13.3) 3 (37.5)	1.78 (0.67-4.74) 0.89 (0.27-2.95) 0.48 (0.14-1.71) 0.36 (0.07-1.71) –	0.20 0.85 0.27 0.18 –
Highest Parent or Guardian Degree Less than Bachelor’s Degree Bachelor’s Degree Post-Graduate Degree	22 (27.2) 18 (22.2) 41 (51.6)	8 (36.4) 4 (22.2) 9 (22.0)	1.66 (0.75-3.68) 1.01 (0.36-2.86) –	0.22 0.98 –
Federal Assistance use when <18 Yes No	27 (33.3) 54 (66.7)	8 (29.6) 13 (24.1)	1.23 (0.58-2.60) –	0.59 –
URiM Status, Sex, Discrimination Experience and ACEs				
Non-modifiable Risk Factor	Number with ACEs ≥3 (%)		Unadjusted Risk Ratios (95% CI)	p-value
Representation in Medicine by Race or Ethnicity ORiM URiM	19 (27.1) 7 (63.6)		– 2.34 (1.30-4.23)	<0.05
Sex Male Female	4 (14.2) 21 (39.6)		– 2.77 (1.06-7.29)	<0.05
Discrimination Experience No Yes	6 (11.8) 15 (50.0)		– 4.25 (1.85-9.77)	<0.01

